# Influence of Pathogens and Mechanical Stimuli in Inflammation

**DOI:** 10.3390/bioengineering6020055

**Published:** 2019-06-25

**Authors:** Israr B.M. Ibrahim, Ramana Pidaparti

**Affiliations:** School of ECAM, College of Engineering, University of Georgia, 200 D. W. Brooks Drive, Athens, GA 30602, USA; israr@uga.edu

**Keywords:** inflammation, cell communication, network

## Abstract

Inflammation is a process driven by underlying cell-cell communication and many other factors. In this study, a model of cell-cell communications was proposed to study factors driving the inflammation time course. Analyses of inflammations that are driven by the combined effects of strain (mechanical stimuli) and/or pathogens are considered in this paper. An agent-based model was employed to simulate inflammation where macrophages and fibroblasts influence each other through cell signaling cytokines that diffuse and spread in the tissue space. The communication network of macrophages and fibroblasts was then inferred and its network model (termed TE network) was generated and analyzed. The results suggest that factors driving inflammation time course can be discriminated by the characteristics of TE networks. Inflammation driven only by pathogens has certain TE network characteristics indicating slower and lower information exchange among cells. Multiple stimuli can help to maintain sufficient information exchange among cells, which is beneficial for inflammation resolution. The TE network captures the unfolding of the innate immune system over time, and the history of pathogens invasion. The resulting network leads to an improved understanding of the resilience of the system to future pathogen invasion.

## 1. Introduction

Inflammation is seen as the cause of many diseases, such as cancer [1,2], autoimmune conditions, atherosclerosis and infections [3], and is a major factor in aging [4]. Hence, developing mathematical analyses of inflammation dynamics is important in order to advance understanding of its underlying mechanisms. Computational models of inflammation [5] ranging from PDE to agent-based models have been employed to study various cases such as the mechano-sensitive response in tissue [6,7], bacterial infection and healing in a model lung environment [8], inflammation induced by particulate matter inhaled into the lung [9], and patterning found in pulmonary [10] and liver fibrosis [11]. 

Inflammation is an example of collective dynamics and is a non-linear process, where many cells and events interact after perturbation to reach equilibrium. Perturbation can occur by over-stretching of tissues [12] or invading foreign matter [9]. There are many factors driving the inflammation time course. Hence, it is important to be able to distinguish the dominant factor(s) influencing its time course in order to improve diagnosis. 

The innate immune system is the first line of defense of the human body. It is less sophisticated than the adaptive immune system as the latter is able to learn and store patterns of disease. However, the innate immune system may also have a memory capability. This memory might play an important role in improving the resilience of the immune system. Resilience in the immune system is defined as the ability of the system to sustain periodic perturbation and adapt to accommodate future attacks [13]. 

Inflammation can be seen as a result of collective information sharing between cells. Motile cells such as macrophages and fibroblasts play a main role in inflammation initiated by the innate immune system. Signaling cytokines regulate their locomotion, activation and interactions. In this study, the inflammation dynamics are examined through the analysis of cell-cell communication. Cell-cell communication is modeled as a network of information exchange between individual cells. It is hypothesized that the structure of these networks and their evolution corresponds to inflammation dynamics, and hence, they can be used to discriminate inflammation dynamics based on factors driving the process. In addition, these networks may be the root of the resilience of the system to invasion.

**Figure 1:**
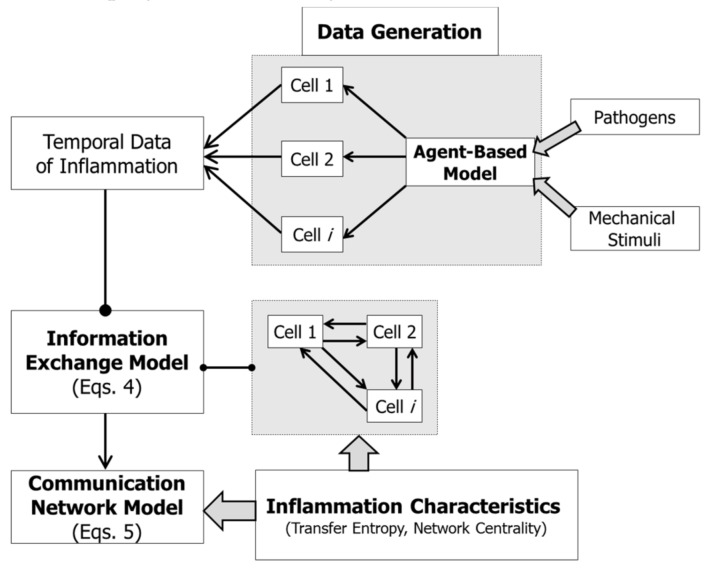
Informatics methods employed in this study to investigate information exchange in inflammation.

The objective of this study is to investigate if information exchange between cells may explain the inflammation time course and resilience of the innate immune system. In order to quantify information exchange occurring during inflammation, it is important to capture the temporal processes associated with inflammation. In this study, simulated data gathered from an agent-based model was used. Information exchange between a pair of individual temporal processes was then quantified, and a network of relation between these processes was generated. Relations between inflammation time course and evolution of network structures were then investigated. Figure 1 illustrates the procedures employed in this study.

## 2. Materials and Methods 

### 2.1. Physiology of Inflammation

Inflammation is a response to stimulation by invading pathogens or endogenous signals such as damaged cells that results in tissue repair or pathology. While it is known that pathogens trigger inflammatory response, tissue strain has been known to activate macrophages in the absence of pathogens [12,14]. Activated macrophages release pro-inflammatory cytokines that will damage epithelial cells, and initiate fibroblast signaling and the wound healing process. Both pathogens and strains may co-exist and drive inflammation. Signaling cytokines signal cells to move across the space according to their concentration gradient (chemotaxis). Chemotaxis guides the macrophages to locate pathogens and fibroblasts to the injury location. The wound healing process leads to fibrosis around the injury site. Fibrosis is the site of elevated stiffness and may cause further damage to the surrounding epithelial cells. 

Based on this description, the macrophage and fibroblast are correlated physically. Hence, it can be inferred that their correlated dynamics correspond to the course of inflammation. These correlated dynamics are quantified through an information exchange (Section 2.3). 

### 2.2. Agent-Based Model for Inflammation

An agent-based model was devised to simulate collective dynamics of cells during the course of inflammation. The core principle of the model is that motile cells (macrophages and fibroblasts) start as random walking agents that also release cell-signaling cytokines such as TNF (pro-inflammatory cytokine) and TGF (anti-inflammatory cytokine). The signaling cytokines spread through diffusion and alter the locomotion of motile cells. Thus, information exchange occurs through diffusion and its speed depends on the diffusion rate.

**Figure 2:**
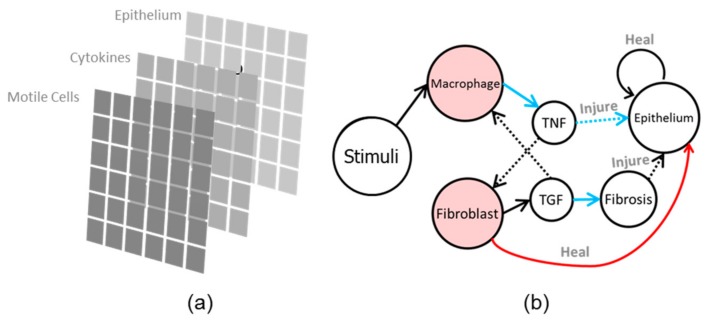
(**a**). Multi-layers structure of the agent-based model; (**b**) illustration of interaction between agents in the model.

The agent-based model has three building blocks: Epithelial cells, motile cells, and a diffusing substance (Figure 2a). Each agent stores a state at a time (such as "alive" or 0) and executes rules to evolve these states. Figure 2b illustrates the interactions between agents in the model. 

#### 2.2.1. Epithelial Cells

Epithelial cells cycle their states between dead (by apoptosis) and alive. Apoptosis depends on the level of TNF [15]. The state transition rules are as follows:

1. All cells are initially alive.

2. A cell has the probability to change its state to death in the presence of TNF in its neighborhood. Higher TNF level significantly increases the chance of apoptosis. We model this likelihood by a beta distribution function. 

3. A cell changes its state to "alive" if at least one fibroblast is present in its neighborhood. This healing process takes place for a specified amount of time, $t_{h}$. After $t_{h}$ time, a neighborhood of healed epithelial cells is randomly assigned a fibrosis site. Fibrosis sites last for $t_{h}$ time and impose damage to epithelial cells by turning a neighboring epithelial cell into a “dead” state. 

4. A cell has the probability to change its state to death if there is a fibrosis site in its neighborhood. The more fibrosis sites there are, the higher the probability. Hence, the probability is determined by $N_{f}/N_{nei}$, where $N_{f}$ is the number of fibrosis sites in the neighborhood (either Moore’s or Neumann’s), and $N_{nei}$ is the total number of neighboring cells. 

5. A cell has $P_{mt}$ probability to change its state to "alive". This is used to model mitosis.

#### 2.2.2. Motile Cells

Macrophages and fibroblasts are motile cells. Biological motile cells move by chemotaxis, and they signal each other through signaling protein (cytokines). Macrophages and fibroblasts are signaled by different cytokines [16]. In our model, macrophages are signaled by TNF (pro-inflammatory cytokine) and fibroblast by TGF (anti-inflammatory cytokine). Macrophages are activated by the presence of strain, and release TNF. Fibroblasts are activated by the presence of TNF, and release TGF. State transition rules are as follows:

1. Movements (random-walk): Motile cells randomly move to an adjacent location on the grid for each time step. The random-walk is biased and the probability weights are determined from the associated cytokine’s value in the cell’s Neumann’s neighborhood. A macrophage’s random-walk bias is determined by values of TNF level obtained from the TNF grid in the Neumann’s neighborhood of the said macrophage’s site. Hence, when no cytokine is present, motile cells movement is a non-biased random walk, with the only interaction being collision avoidance where each motile cell avoids occupying the same space as the others. 

2. Activation: Macrophages are activated by the presence of strain and pathogens, and when macrophages release pro-inflammatory cytokine (TNF). TNF release is probabilistic according to the level of TGF [14]. Fibroblasts are activated by the presence of pro-inflammatory cytokines, and release anti-inflammatory cytokine (TGF). TGF release is probabilistic according to level of TNF [17]. 

3. Cytokine secretion: Each motile cell releases cytokine stochastically. This probability is defined by the cytokine’s value located on the same location as the motile cell. The probability of a macrophage releasing TNF is determined by the absence of TGF on its location. The probability of a fibroblast releasing TGF is determined by the absence of TNF on its location.

#### 2.2.3. Cytokines Diffusion

The cytokines in this model are assumed to be diffusing substances. There are two types of cytokines: The pro-inflammatory one (termed TNF) and anti-inflammatory cytokine (termed TGF). The rule is essentially a numerical solution of the diffusion equation:

(1) $$\frac{d\phi }{dt}=D\cdot \nabla^{2}\phi -K\cdot \phi$$

where $\nabla =\frac{\partial }{\partial x}+\frac{\partial }{\partial y}$ is Laplacian in two dimensions, ϕ is cytokine’s concentration, D is the diffusion constant that controls the spread rate of cytokine, and K is the dissolution constant that controls the decaying rate of the cytokine. Typically, D is>K.

#### 2.2.4. Pathogens and Strain

Pathogens are modeled as random walking agents that only avoid collision with motile cells and other pathogens. Pathogens replicate periodically to simulate the inhalation and the changing nature of pathogen concentration in the environment. Pathogen count (Pc) throughout the simulation is modeled by,

(2) Pc(t)=A sinα·t

where t is the simulation time, A is the amplitude, and α is the rate constant. The pathogen’s size was assumed to be uniform.

Strains are assumed to be a field in the tissue space. Since the time frame of strain and inflammation differs greatly, an average stimulus value was applied to every grid in the agent-based model.

### 2.3. Information Exchange Model

To quantify the information exchange in inflammation, it is necessary to consider the temporal process that can be gathered/observed. Migration properties such as directional persistence [18] have been used in analysis of cell collective dynamics. In this study, the information exchange between macrophage and fibroblast was analyzed on the basis of their change of angle (which can be experimentally collected). The reasoning is that if there is an information transmission from a macrophage to a fibroblast, then it will alter the directionality of the said fibroblast’s migration. The direction of a cell can be represented as the angle between the current and previous path taken by a cell, i.e.,

(3) $$\theta_{t,t-1}=\cos^{-1}\frac{m^{t}\cdot m^{t-1}}{\left|m^{t}\right|\left|m^{t-1}\right|}$$

where $m^{t}$ is the vector of path of a cell at time t. We denote this angle as $M^{t}$ and $F^{t}$ for macrophage and fibroblast, respectively. The transfer entropy, $T_{F\to M}$, between $M_{t}$ and $F_{t}$ can be defined as mutual information exchange between the two random processes conditional on a previous instance of $M_{t}$. It is expressed as,

(4) $$T_{M\to F}=\underset{M_{t}^{i}}{\sum }\underset{M_{t-1}^{j}}{\sum }\underset{F_{t-1}^{k}}{\sum }p\left(M_{t}^{i},M_{t-1}^{j},F_{t-1}^{k}\right)\times \frac{p\left(M_{t}^{i}|M_{t-1}^{j},F_{t-1}^{k}\right)}{p\left(M_{t}^{i}|M_{t-1}^{j}\right)}$$

where $p\left(M_{t}\right)|M_{t-1},F_{t-1}$ is the probability of $M_{t}$ given $M_{t-1}$ and $F_{t-1}$. The probability of $M_{t}$, $M_{t-1}$ and $F_{t-1}$ is calculated by generating joint probability densities using binning specifications between 0.001 and 0.01. In this case, the transfer entropy can be thought of as the reduction in uncertainty of predicting the direction of a fibroblast at time step t given its previous direction (at t−1) and direction of a macrophage. 

### 2.4. Communication Network Model

To model cell-cell relations, a network model is used. The network consists of a set of nodes representing each cell in a simulation, with the edges representing relations between each node. A pair of nodes may or may not be connected by an edge. To determine whether a pair of nodes is connected, transfer entropy, $T_{M\to F}$, was used. Transfer entropy here is used as a measure of information exchange between two processes, in this case information exchange between a pair of cells. 

**Figure 3:**
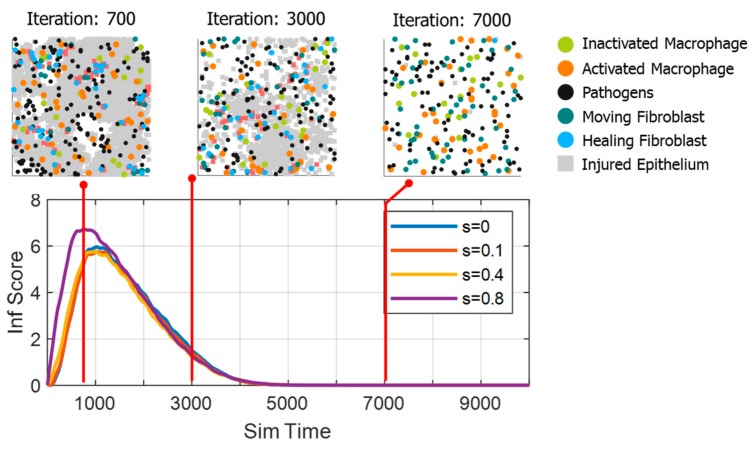
Inflammation time course for cases of combined effect of the strain and pathogens in the tissue. The inset shows enlarged view of time course when s=0.1. A sample of constituent distribution is also shown with color codes as follows: Blue = fibroblasts, green = macrophages, red = activated macrophages, black = pathogens, grey = dead epithelial cells (injuries).

To determine an edge between a pair of nodes, an algorithm was used. Suppose a network $G_{MF}$ starts with edges connecting every node representing macrophage (M) and fibroblast (F), as illustrated in Figure 3. The edge then is iteratively removed according to $T_{M\to F}$ value of a pair of nodes as calculated by Equation (4). If a pair of macrophage and fibroblast yields $T_{M\to F}$ below a certain threshold $T_{C}$, then an edge between them is not included in $G_{MF}$. This rule can be expressed as,

(5) $$f\left(M_{i},F_{i}\right)=\{\begin{array}{c} \left\{M_{i},F_{i}\right\}\;\in \;\;G_{MF},\;\;\;T_{M_{i}\to F_{i}}>T_{C}\\ \{M_{i},F_{i}]\;\notin \;\;G_{MF},\;\;\;T_{M_{i}\to F_{i}}\leq T_{C} \end{array}$$

where $\left\{M_{i},F_{i}\right\}$ denotes an edge between two nodes, $M_{i}$ and $F_{i}$. The generated network is termed the transfer entropy (TE) network throughout the rest of the paper.

We derived $T_{C}$ from the estimated distribution of $T_{M\to F}$. Since $T_{M\to F}$ is calculated for every pair of macrophage and fibroblast, distribution of $T_{M\to F}$ is then estimated and the most likely values of $T_{M\to F}$ are used for the edge generation criterion. A range of values is defined based on the first and third quartiles of the $T_{M\to F}$ distribution as the criterion of edge inclusion and to account for asymmetry of the distribution.

To identify the dominant actor in the TE network, we estimated the eigenvector centrality. A node that is connected to many other high centrality nodes will score high in eigenvector centrality. Thus, in terms of information flow, nodes with higher centrality accumulate their influence relative to the rest of the nodes in the network. Eigenvector centrality $c_{i}$ of a node i is described by [19],

(6) Ac=λc

where A is the adjacency matrix of the graph describing the network, and λ is the largest eigenvalue of A. Since the TE networks can be conveniently divided into macrophage (M) nodes and fibroblast (F) nodes, the centrality values of M nodes are termed as Mc and those of F nodes are termed Fc in this paper.

## 3. Results and Discussion

### 3.1. Data Generation

Several simulations with strain as a stimulus were carried out, each with 10 trials for the case studies. The strain values (S) were varied from 0 (no strain), 0.1 (low strain), 0.4 (medium strain) and 0.8 (high strain) in the case studies. Pathogen replication was performed according to Equation (2) with A=1 and α=25. Simulations were carried out by combining the effect of four strain cases and pathogens (Case A), and the effect of the strain alone (Case B).

### 3.2. Comparison of Inflammation Time Course

Figure 3 shows the inflammatory time course in terms of an inflammation score. Inflammation scores were obtained from the number of dead epithelial counts (injury) divided by 1000. A general pattern of inflammation time course was observed, where the inflammatory reaction rose quickly (inflammation phase) followed by a recovery phase [20]. During the inflammation phase, the tissue injury occurred rapidly because the pro-inflammatory cytokines (TNF) were released by activated macrophages (either by strain or pathogens). After some simulation time, the pro-inflammatory cytokines also triggered fibroblasts to release anti-inflammatory cytokines that recover the tissue. 

In Case A (Figure 3), increasing the strain from 0 to 0.1 led to a slight decrease in inflammation score amplitude, and a quicker inflammation phase (shifts of inclining trend to the left). However, increasing the strain further to 0.8 led to a more significant change in amplitude and inflammation phase duration 

**Figure 4:**
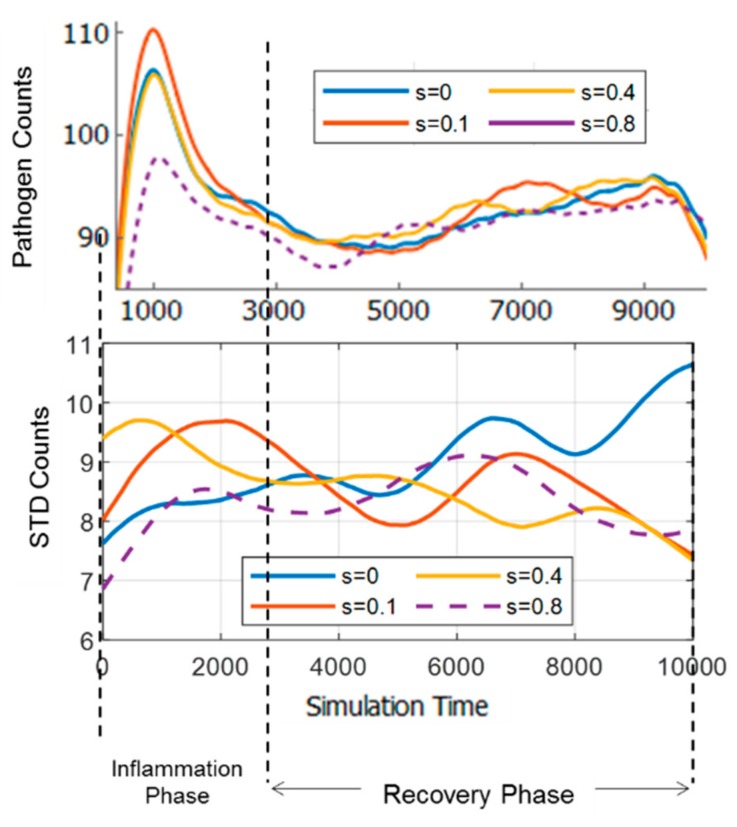
**Top**: Pathogen counts (mean of 10 simulation trials) plotted against simulation time. **Bottom**: Standard deviation of pathogen counts from 10 simulation trials for each case.

The pathogen counts (the means of 10 simulation trials and standard deviations) time course for each strain level is shown in Figure 4 (Top). It can be observed that the time courses are in general suppressed in the middle of the inflammation. Moreover, it can also be observed from Figure 4 that at the onset of the recovery phase, pathogen counts declined. However, the pathogen counts may keep declining in most of the 10 trials, or may change course and incline again in most of the trials. This can be visualized by plotting the standard deviation of pathogen counts (Figure 4). It can also be observed that the standard deviation may diverge or converge as simulation time progresses. It can be seen in the case of s=0 (no strain), the pathogens count tended to diverge. On the other hand, when the strain was increased to 0.1 and 0.4, the pathogen counts tended to diverge at first, and then converged at the end of the simulation, indicating a stable outcome of the inflammatory response. However, when the strain was increased further to 0.8, the convergence at the end became less pronounced. These results indicate that strains, although causing inflammatory response themselves, may drive the pathogen-induced inflammation in different courses. 

### 3.3. Dynamics of Information Exchange between Macrophage and Fibroblast

**Figure 5:**
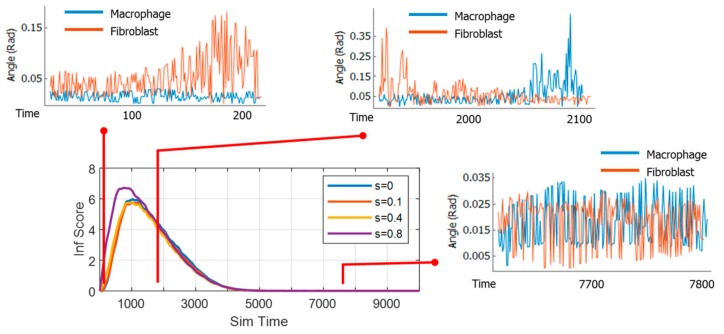
Sample data (above) of macrophages (red) and fibroblasts (blue) tracks from which change of directions (Equation (3)) were calculated and used to infer information exchange between cells (Equation (4)).

The results from the previous sections indicated that each case of inflammation may have its own underlying collective dynamics of immune cells, which may explain why certain inflammation progresses the way it does. In this section, the inflammation time course is explained through cell-cell relations as described by the TE network. The macrophage and fibroblast change of directions were extracted and calculated according to Equation (3). Samples of these data at various locations in the time course are shown in Figure 5. The TE networks were generated by the algorithm presented in Equation (5). The centrality values for fibroblast nodes (Fc) and macrophage nodes (Mc) were calculated according to Equation (6).

**Figure 6:**
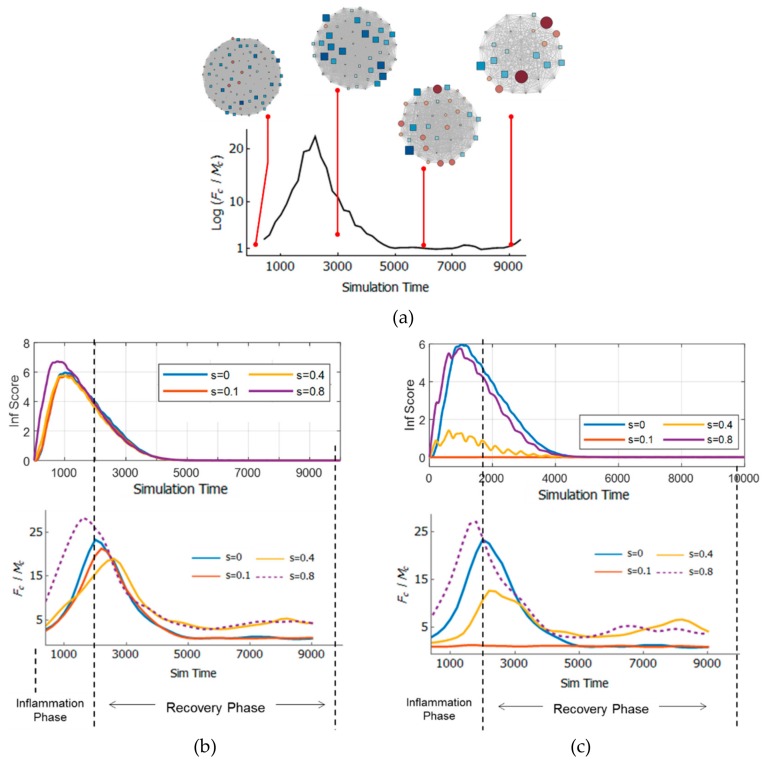
(**a**) Dynamics of the transfer entropy (TE) network with respect to time; (**b**) dynamics of the TE network for inflammation cases with combined effects of pathogens and strain, and (**c**) only pathogens.

Figure 6a shows the TE network time course as an evolution of Fc/Mc with time for s=0. It can be observed from the visualization that at first, the macrophage (red) and fibroblast (blue) nodes sizes are comparable. As time progresses, the fibroblast node size increased at some point, indicating increased centrality in the network as shown in Figure 6b. As recovery phase progressed, the node size of the fibroblast reduced and macrophage node size increased near the end of the simulation. 

To quantify the TE network time course, the ratio (Fc/Mc) of the fibroblast node’s centrality (Fc) to macrophage’s node centrality (Mc) was used for subsequent analyses (refer to Section 2.4). High Fc/Mc indicates fibroblasts dominate the system at a time point. Figure 6b and c shows Fc/Mc compared with the inflammation time course. 

In Case A (Figure 6b) The peak of Fc/Mc can be observed at a point close to the onset of the recovery phase. It can be observed that the dynamics of TE networks for lower strain (s=0 and s=0.1) and higher strain (s=0.4 and s=0.8) can be clearly distinguished. For s=0.8, the peak of Fc/Mc shifted to the left, indicating that increasing the strain accelerated the peak of Fc/Mc. It can also be observed that higher strain (s=0.4 and s=0.8) leads to higher Fc/Mc at the tail of the time course (toward the end of the inflammation). 

In Case B (Figure 6c (Top)), only the strain affected the collective dynamics of the immune cells. At s=0.1, there is little to no inflammation. When the strain was increased to 0.4 and 0.8, a similar time course as in Case A was observed, however with less amplitude and rougher courses. In Case A, this rough time course was smoothed out by a response triggered by pathogens, which diluted the tissue space with pro-inflammatory cytokines. These results indicate that each condition may have unique underlying collective dynamics.

Figure 6c (Bottom) shows the TE chart for Case B. As can be seen, for s=0.1, the inflammation score is flat and Fc/Mc is erratic and lowest, indicating little to no cell-cell communication because there is little inflammatory response. When the strain was increased, the Fc/Mc peaked at the initial recovery phase as seen in previous cases shown in Figure 6b. 

**Figure 7:**
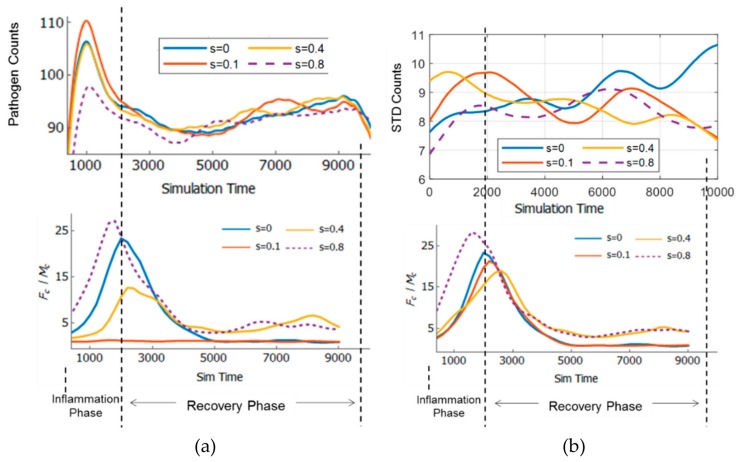
Comparison of TE network dynamics to (**a**) pathogen counts time course, (**b**) SD (standard deviation) of pathogen counts.

Figure 7a and b show the comparison of the TE network dynamics with pathogen counts time course and standard deviation (SD), respectively. As can be seen in Figure 6a, there is no noticeable relation between the time course of pathogen counts with the network structure. However, it can be seen in Figure 7b that the SD of pathogen counts time course for s=0 cases has an increasing trend, while the others have the opposite trends overall. The SD of pathogen counts measures the stability of the simulation (whether the inflammation time course tends to diverge or converge based on the number of simulation trials for each case). It can also be seen that s=0.1 and s=0.4 have similar dynamics as the SD increased early in the time course (up to 3000 simulation time), while for s=0.8, the SD increased gradually (following the trend of s=0) before decreasing towards the end.

### 3.4. Effect of Strain and Pathogen to Cell’s Information Exchange

**Figure 8:**
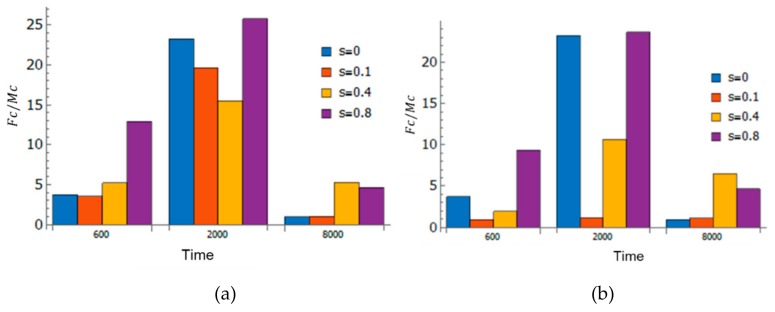
Fc/Mc at a discrete time of 600, 2000, and 8000 for (**a**) cases with pathogens, and (**b**) cases with only the strain.

Inflammation as a process depends on many factors. In this study, it is driven by two main factors: Strain and pathogens. These factors and their combination impacts the information exchange between cells differently. Figure 8 demonstrates the impact of altered information exchange through these networks. Early in the inflammation time course (at time = 600 in Figure 8), the network’s nodes have similar centralities (represented by node sizes as shown in Figure 6a). As time progressed (at time = 2000 in Figure 8), fibroblasts dominated the collective dynamics at the onset of the recovery phase as indicated by the large Fc/Mc ratio shown in Figure 6 (and blue node’s size as shown in Figure 6a). Eventually (at time = 8000 in Figure 8), fibroblast domination declined and shifted to macrophages dominating the collective dynamics as indicated by the low Fc/Mc ratio. These results indicate that the inflammation driven by pathogens and strains can be distinguished based on their TE networks.

### 3.5. Resilience of the Innate Immune System

It can be observed from Figure 8 that the higher strain (s=0.4 and s=0.8) resulted in the higher Fc/Mc at the end of the recovery phase. It indicates that cell-cell information exchanges were still maintained by the presence of the strain, which resulted in more stable suppression of pathogen counts (lower SD in Figure 7b). The latter can be seen as resilience of the immune system to invading foreign matter. Hence, it can be concluded that inflammation with combined factors may increase resilience of the tissue (one stimuli may result in more resilience toward defense against other stimuli) through increased cell-cell communications and is beneficial in the long term, although the inflammation scores were high early in the time course.

### 3.6. Adaptivity of Innate Immune System

The innate immune system does not have memory and adaptive mechanisms. However, the process itself may have memory (similar to hysteresis). This memory may emerge from the dynamics of information exchange between cells, which is represented by TE networks in this study. For each case of stimuli, repeating the simulation may result in increasing or decreasing pathogen counts toward the end of the inflammation. However, as can be seen in Figure 7b, for cases with stimuli (s>0), the standard deviation (SD) of ten simulation trials indicated high SD early in the time courses, and declining trend toward the end of time course. It indicates that the early inflammation time courses were divergent and more uncertain in terms of its course, and became more convergent, more stable toward the end. The interpretation is that the system at first was vulnerable, but as time progresses, it became more adept at suppressing pathogens. Thus, it implies that the system exhibits memory that leads to adaptation in pathogen suppression.

This adaptive feature may also be explained through the evolution of cell-cell information exchange dynamics (TE networks). Early in the time course, the TE networks accumulated the history of the process in order to tackle the inflammatory response caused by stimuli. This is illustrated through dynamic TE network structures shown in Figure 6a. As time progresses, the system experimented with various TE network structures depending on the initial and environmental conditions. The TE networks stored the history of the process in order to increase the readiness and resilience of the system to future invasions of pathogens, which are unpredictable spatially (but periodic temporally), given the re-randomization nature of the simulation. The final result is increased resilience as is indicated by declining SD of pathogen counts for cases where the strain exist (s>0). However, when s=0, the opposite applies as the standard deviation increased steadily during the inflammation. Hence, the combined effect of the strain and pathogens is necessary to induce memory and adaptation in the system.

### 3.7. Limitations

This study focuses on inflammation initiated by the innate immune system. While other cells such as neutrophil are part of the innate immune system, the two types of cells selected in this study better reflect the role of cells in the innate inflammatory response: Cells involved in phagocytosis and signaled by pro-inflammatory cytokine (e.g., macrophages, neutrophils) and cells involved in wound healing and signaled by anti-inflammatory cytokine (fibroblasts).

The simulation in this study was limited to cases where the pathogen’s size variation is not too large and can be modeled as uniform in size, hence the steric effect was minimal and not considered. 

It was assumed that locomotion of cells was influenced mainly by cytokine gradients in this study. Thus, the effect of strain on locomotion was not included. In addition, the space in the simulation was defined by a grid. Therefore, the angle of the cell’s locomotion was limited to eight directions of Moore’s neighborhood (corresponding to eight wind directions in a compass). Future studies may consider including the effect of additional directions.

## 4. Conclusions

Inflammation is a process driven by many factors as well as underlying cell-cell communication. In this study, a model of cell-cell communications was proposed to study factors driving inflammation time course. Analyses of inflammations that are driven by the combined effects of strain and/or pathogens are considered in this paper. An agent-based model was employed to simulate inflammation where macrophages and fibroblasts influence each other through cell signaling cytokines that diffuses and spread in the tissue space. The communication network of macrophages and fibroblasts was then inferred and its network model (termed TE networks) was generated and analyzed. 

The results suggest that factors driving inflammation time course can be discriminated by the characteristics of TE networks (network centrality). Inflammation driven only by pathogens has certain TE network characteristics indicating slower and lower information exchange among cells. Multiple stimuli can help to maintain sufficient information exchange among cells, which is beneficial for inflammation resolution. 

The TE network might also explain the memory of the innate immune system as a process. As inflammation progresses, the TE network collects the history of pathogen invasion that is noisy but periodic. The resulting network leads to an improved resilience of the system to future pathogen invasion.
